# Pork carcass fabrication economics: drivers of profitability and an explanation of costing models

**DOI:** 10.1093/tas/txag024

**Published:** 2026-02-26

**Authors:** B M Bohrer

**Affiliations:** Department of Animal Sciences, The Ohio State University, Columbus, OH, 43210, United States

**Keywords:** cost absorption, fabrication economics, meat processing, meat systems, pork industry

## Abstract

Pork carcass fabrication is a central determinant of value realization within packing and processing systems, translating biological variation in carcass weight and composition into economic outcomes under dynamic market conditions. The objective of this review is to synthesize current knowledge on the economic drivers of pork carcass fabrication, with a specific focus on the interactions among carcass characteristics, fabrication strategies, and value realization. Regional differences in cutting specifications and market orientation are discussed as key factors shaping primal yields, market allocation, and value distribution across domestic and export channels. The economic contributions of primals, subprimals, trim, fat, and by-products are examined in the context of wholesale pricing signals, carcass merit programs, and packer-specific specifications that link production decisions with downstream processing requirements. Fabrication strategies, including depth of fabrication and primal-specific cutting decisions, are evaluated with respect to yield optimization, labor and packaging costs, and market flexibility. The influence of carcass weight and composition on fabrication efficiency, trim generation, and fixed cost allocation is highlighted, illustrating trade-offs between biological performance and processing constraints. Technological advancements, including instrument grading, automation, and data integration, are reviewed for their role in improving yield prediction, carcass sorting, and operational consistency, while emerging tools such as predictive modeling are identified as promising approaches for managing variability and economic risk. Price volatility, biological variability, and supply chain disruptions are identified as persistent challenges to fabrication economics, underscoring the need for resilient and adaptable processing systems. Beyond economic performance, fabrication decisions are discussed in relation to labor welfare and sustainability outcomes. Collectively, this review emphasizes that optimal pork carcass fabrication is achieved through the strategic integration of biological inputs, economic signals, and operational capabilities. Improved data transparency and collaboration between industry professionals are essential to develop integrated biological-economic frameworks that enhance value realization and long-term sustainability across the pork supply chain.

## Teaser

Pork carcass fabrication decisions quietly shape profitability across the entire supply chain, from primal yields to labor efficiency and market access. This review explores how carcass characteristics, fabrication strategies, and emerging technologies interact with market dynamics to influence economic outcomes in the modern pork industry.

## Introduction

Carcass fabrication is a critical determinant of carcass value and represents one of the most economically influential stages in the packing and processing sectors of the pork industry. By definition, carcass fabrication encompasses the conversion of whole carcasses into merchandized pieces which can include primals, subprimals, trim, fat, and by-products (such as bones, jowls, tails, and feet). With narrow profit margins throughout the pork industry, fabrication efficiency and decisions related to the extent of fabrication play a pivotal role in capturing carcass value and maintaining competitiveness (Boland et al. 1995).

Decisions made during fabrication directly influence profitability across the pork supply chain, affecting labor requirements, product yields, processing efficiency, and market flexibility (Lorenzen et al. 1996a, 1996b). Specifically, decisions regarding the depth of fabrication, cut specifications, and product form determine how carcass value is distributed among domestic and export markets and how effectively packing plants respond to changing market signals. These decisions also provide influential feedback to production systems through pricing mechanisms and carcass merit programs, reinforcing alignment between biological performance and downstream economic outcomes.

The objective of this review paper is to synthesize current knowledge on the economic drivers of pork carcass fabrication, with a specific focus on the interactions among carcass characteristics, fabrication strategies, and value realization. Outcomes relevant to packers, processors, and integrated production systems are emphasized, highlighting how fabrication decisions translate biological variation into economic performance and identifying areas where improved integration of biological and economic data may enhance strategy and value across the pork supply chain.

## Overview of pork carcass value formation

### Carcass fabrication in different regions of the world

Merchandizing orientation, defined as the practice of producing meat products that fulfill the specifications and value signals of specific customers or markets, plays a critical role in determining the relative value of carcass components. Markets around the world often prioritize different cuts from a pork carcass, some of which may influence the cutting specifications of primal pieces while others may influence how said primal pieces are further fabricated into merchandized cuts (Boland et al. 1995). In North America, the Institutional Meat Purchase Specifications (IMPS), which is maintained by The Meat Institute along with the American Meat Science Association and the USDA Agricultural Marketing Services, provides detailed, standardized descriptions of cutting specifications that define the anatomical origin, dimensions, and attributes of merchandized meat cuts so buyers and sellers can trade with confidence and clarity (The Meat Institute 2025). The result of IMPS cutting specifications can be observed in Table 1. In Europe, the United Nations Economic Commissions for Europe (UNECE) Standard for Porcine Meat—Carcases and Cuts provides standardized cutting specifications and identification for pork primal and subprimals in a similar manner to the North American IMPS (UNECE, 2008). Other documents for cutting specifications include the China National Standard for Fresh and Frozen Pork (Standardization Administration of the People’s Republic of China 2019), the Guide to Meats in Spain (Interporc Spain, 2020), and the Australian Pork Training Manual (Australian Pork 2023).

**Table 1:** Assumptions for yield of primal cuts.^a^^,^^b^

Primal	Percentage of Carcass (%)
**Loin**	25.17
**Butt**	10.16
**Picnic**	11.37
**Sparerib**	4.81
**Ham**	24.59
**Belly**	16.28
**Jowl**	1.45
**Neckbones**	1.69
**Tails**	0.15
**Front feet**	0.92
**Hind feet**	1.23
**Cut loss**	2.18

a *Source*. USDA Agricultural Marketing Service User’s Guide to USDA’s Pork Carcass Cutout (2026b).

b Calculations based on a 97.5 kg carcass, 55–56% lean, and 14–18 mm of backfat thickness.

Markets around the world may deviate from the IMPS or UNECE standards and ascribe to a variety of different cutting specifications. Major differences in cutting specifications include those related to separation point of the shoulder and the loin/belly and separation point of the ham and the loin/belly (Ray and Cravens 2002). In general, pork produced in North American markets separate the shoulder and the loin/belly between the first and second rib or between the second and third rib; whereas, pork produced in (or destined for) Asian, European, or South American markets generally separate the shoulder and the loin/belly between the fourth and fifth rib. Additionally, pork produced in North American markets generally include the sirloin with the loin primal; whereas, pork produced in (or destined for) some Asian, European, and South American markets may include the sirloin with the ham primal. These cutting specifications have noticeable influences on primal weights, dimensions, and yields as illustrated by Ray and Cravens (2002), who provided a comparison of cutting yields between “American-style” and “Japanese-style” specifications.

Several recent research studies have evaluated alternative fabrication strategies for pork carcasses. Bryan et al. (2018) reported that adjusting pork carcass fabrication strategies to export-oriented specifications (export-oriented defined as Asian markets) shifted yields from loins and bellies to higher-value shoulders, hams, and novel cuts, resulting in equal or greater overall carcass value compared with conventional IMPS under several different pricing scenarios. Additionally, Metz et al. (2024) reported that heavy-weight pork carcasses (defined as heavy carcasses weighing 116 kg to 126 kg and very-heavy carcasses weighing 134–144 kg) had heavier primal and subprimal weights compared with average-weight pork carcasses (defined as carcasses weighing 99–109 kg) without substantially altering weight distribution throughout the carcass. Furthermore, Metz et al. (2024) suggested that when combined with alternative fabrication specifications, heavy-weight and very heavy-weight carcasses produced sufficiently sized novel shoulder cuts, such as the *serratus ventralis* and *triceps brachii* muscles, that merit consideration as retail products.

### Components of carcass value

**Figure 1:**
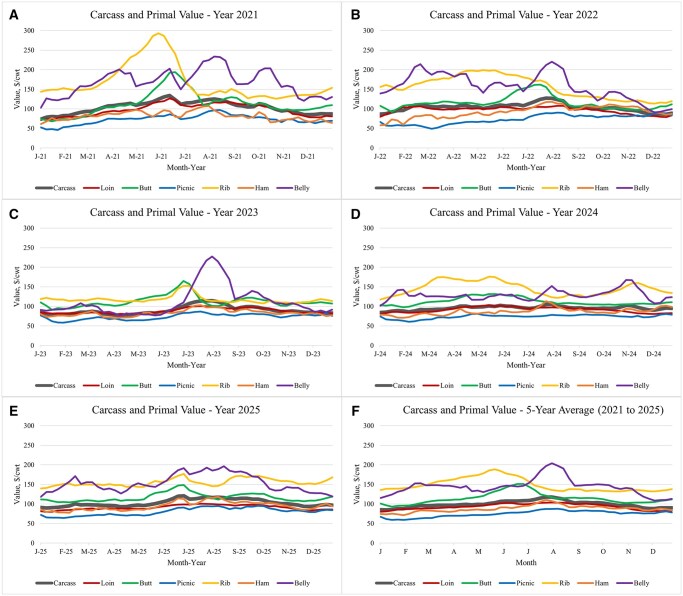
Weekly carcass and primal value for the most recent 5-year period (2021–2025; source: USDA agricultural marketing service weekly National Carlot Report, 2026a).

For the purposes of this review, the United States (and the economic and statistical reports provided by the USDA) serves as the primary model for discussion and illustrative examples. Economic value of a pork carcass is derived from the combined contributions of individual primals, subprimals, trim, fat, and by-products, each of which varies in market demand, yield, and pricing structure (as shown in the USDA Agricultural Marketing Service Weekly National Carlot Report 2026a). Traditionally, high-value primals such as the belly, rib, and butt are more variable and account for a disproportionate share of total carcass value, while low-value primals such as the picnic, loin, and ham contribute to the consistent nature of carcass value throughout a given year (Fig. 1). A detailed user’s guide to pork carcass cutout is available from the USDA Agricultural Marketing Service (USDA Agricultural Marketing Service 2026b). Within this guide, detailed information related to the market analysis and price discovery are reported, while the assumptions and calculations used for the aforementioned USDA Weekly National Carlot Report are described.

While much attention has been garnered for the relationship between lean hog prices and the calculation of carcass cutout values (Tonsor and Parcell 2025), the focus of this review is to provide insight into packer and processor dynamics affecting decisions related to fabrication strategies and value realization.

Lean yield and fat trim further shape carcass value by affecting both saleable meat yield and processing efficiency (Lorenzen et al. 1996a; Pringle and Williams 2001). Greater lean yield generally enhances carcass value for the packing industry by increasing the proportion of marketable cuts, whereas excessive fat trim may reduce value and/or require diversion to lower-value market channels. However, fat also contributes positively through its use in processed products and rendering (Cannon et al. 1995). Carcass by-products (*e.g.*, bones, skin, tails, and feet) and non-carcass by-products (*e.g.*, organ meats, heads, and blood) provide additional revenue streams that collectively contribute to value recovery for packing plants (Marti et al. 2012; Seong et al. 2014). Similar to primals, subprimals, trim, and edible fat, by-products vary in market demand, yield, and pricing structure (as shown in the USDA Agricultural Marketing Service Weekly By-Product Drop Value Report 2026c).

### Market signals and pricing systems

Market signals transmitted through pricing systems play a critical role in shaping pork carcass composition and fabrication strategies. Grid pricing and carcass merit programs are designed to reward producers, or production systems in the case of vertically integrated systems, for carcasses that align with packer and market preferences, typically emphasizing lean yield, optimal carcass weight, and consistency (Hayenga et al. 1985; Keeler et al. 1994; Zhou and Bohrer 2019). These pricing systems directly influence on-farm decisions, such as genetic selection, nutrition, and marketing decisions, by economically incentivizing carcasses that maximize value under prevailing grid parameters. As a result, carcass traits that are not explicitly rewarded (*i.e.*, penalized) may be deprioritized despite potential value in alternative fabrication or emerging markets.

Packer-specific cutting specifications further refine how carcass value is realized by dictating acceptable ranges for carcass weight and carcass composition. Because packers differ in customer base, export exposure, and fabrication capabilities, cutting specifications can vary substantially across firms, leading to differences in how identical carcasses are valued and utilized. Such variability underscores the importance of aligning production systems with the intended packer and their specific market channels, as deviations from preferred specifications can result in discounts or reduced fabrication efficiency, even when carcasses are otherwise free of defects.

Wholesale cut values, including the pork cutout value, serve as a key market signal linking carcass characteristics to downstream demand. Fluctuations in cutout values reflect shifts in consumer preferences, export demand, and seasonal buying patterns, and they influence the relative economic importance of individual primals and subprimals. Consequently, changes in wholesale pricing can alter the balance between traditional and alternative fabrication strategies, as packers seek to maximize returns by emphasizing higher-value cuts. Integrating carcass merit programs with real-time wholesale market signals may therefore improve value across the pork supply chain.

## Fabrication strategies

### Fabrication level and cut specifications

Fabrication strategy is a primary determinant of value recovery in pork processing, as decisions regarding the level and type of fabrication directly influence yield, labor requirements, and market flexibility. Theoretically, processors may market pork as whole carcass sides, primals, subprimals, or fully fabricated case-ready products, with each approach presenting distinct economic trade-offs. Marketing of whole carcass sides minimizes fabrication labor and capital investment but severely limits value optimization and exposure to higher-margin markets. For this reason, medium- and large-sized packers rarely market whole carcass sides. Primal and subprimal fabrication allows processors to better align product offerings with downstream customer specifications, improve price discovery, and selectively allocate raw materials toward the highest-valued uses.

Case-ready and further-processed product strategies represent the deepest level of fabrication and are increasingly used by medium- and large-sized packers to capture additional margin through branding opportunities, portion control, and shifting labor from the retail sector to the processing sector. However, these strategies introduce greater costs associated with labor, packaging materials, inventory management, and shrink, requiring careful margin analysis to ensure incremental revenues exceed added variable and fixed costs. Consequently, fabrication depth is often determined by a processor’s ability to manage labor availability, capital intensity, and demand volatility across product categories.

### Primal-specific fabrication decisions

Approximately one-quarter to one-third of the pork carcass (on a weight-basis) in commercial systems is fabricated into case-ready products, although this proportion varies substantially by processor and market orientation. Retail-focused and vertically integrated operations tend to exhibit greater case-ready utilization due to greater volume and a more unified marketing/branding approach. This, in turn, maintains skilled labor at the packing plant level while shifting it away from the retail level. In contrast, processors oriented toward commodity, foodservice, or export markets typically fabricate a smaller share of the carcass into case-ready formats, reflecting differences in customer specifications, labor availability, and margin structures. Beyond retail case-ready fabrication, a substantial proportion of the pork carcass is directed toward further processing, where value is added through comminution, seasoning, curing, thermal processing, or product assembly into ready-to-prepare or ready-to-eat products. Cuts commonly utilized for further-processed products include hams for cured and smoked items, bellies for bacon, picnics and shoulders for sausages or lunch meat, and trim derived from primals and subprimals for ground and formulated items. Further processing provides processors with flexibility to manage variability in carcass composition, balance supply with demand, and optimize utilization of lower-value or cuts of variable sizes. As a result, fabrication decisions at the packing level are closely linked to downstream processing strategies, as the economic value of individual cuts is often realized not at fabrication, but through subsequent transformation into branded or differentiated products.

Loin fabrication strategies illustrate the trade-off between yield optimization, labor intensity, and market alignment. Bone-in loins provide greater yields and lower labor inputs, while boneless and portion-controlled loins command price premiums but require additional trimming, handling, and quality control. Portion control introduces tighter specifications that increase trim generation, shifting value into secondary product streams that must be effectively monetized to preserve margins. Assumptions derived from the USDA for yield of merchandized cuts from the loin primal are provided in Table 2 (USDA Agricultural Marketing Service 2026b). Using single point in time data, the calculations shown in Table 3 illustrate value captured from various loin cutting strategies. This table can be easily modified as price fluctuations occur during the year but in general, the greatest level of loin fabrication (boneless, center-cut, strap-off loin) has the potential to increase value proposition by approximately 36%. In addition to fabrication intensity, export demand and customer specifications play a critical role in determining optimal loin utilization. Unlike the shoulder/butt, where export markets often drive volume and specification decisions, loin export demand is more variable and highly dependent on cutting specifications. Certain markets require bone-in product forms or specific muscle configurations, which may limit the feasibility of aggressive further fabrication. Consequently, packers must balance domestic retail premiums with export program commitments, as shifts in international demand can materially affect loin cutting strategy and realized value. It is also important to note that while the bone-in center-cut tender-in loin represents one of the highest total-value items derived from the loin primal (behind only to more extensively fabricated options), its overall demand remains relatively low. The elevated value of this item is driven largely by scarcity rather than broad market pull. In other words, it is a high-value but low-volume product. Overproduction relative to demand would likely erode its premium, reinforcing the importance of aligning fabrication decisions with true end-market demand rather than theoretical cut value alone.

**Table 2:** Assumptions for cutting yields of merchandized cuts from the loin primal.^a^^,^^b^

	Loin Cutting Strategy					
*Merchandized components*	Untrimmed Loin Primal (Drop Loin)	1/4” Trimmed Loin	1/8” Trimmed Loin	Bone-in, Center-Cut, Tender-In Loin	Boneless, Center-Cut, Strap-On Loin	Boneless, Center-Cut, Strap-Off Loin
**Untrimmed loin primal (drop loin)**	100.00%	–	–	–	–	–
**1/4” trimmed loin**	–	84.54%	–	–	–	–
**1/8” trimmed loin**	–	–	80.85%	–	–	–
**Bone-in, center-cut, tender-in loin**	–	–	–	52.11%	–	–
**Boneless, center-cut, strap-on loin**	–	–	–	–	32.93%	–
**Boneless, center-cut, strap-off loin**	–	–	–	–	–	28.88%
**Boneless sirloin**	–	–	–	6.90%	6.64%	6.64%
**Blade end**	–	–	–	9.74%	–	–
**Tenderloin**	–	–	–	–	4.04%	4.04%
**Butt tender**	–	–	–	1.07%	–	–
**Backribs**	–	–	–	–	9.13%	9.13%
**Riblets**	–	–	–	–	1.29%	1.29%
**72% trim**	–	0.44%	0.44%	6.22%	12.93%	16.37%
**42% Trim**	–	–	–	4.24%	4.35%	4.95%
**Fat**	–	11.05%	14.53%	11.05%	11.05%	11.05%
**Skin**	–	3.47%	3.47%	3.47%	3.47%	3.47%
**Bone**	–	–	–	4.28%	13.07%	13.07%
**Shrink**	–	0.50%	0.71%	0.92%	1.11%	1.11%

a Assumptions are derived from values reported by USDA Agricultural Marketing Service User’s Guide to USDA’s Pork Carcass Cutout (2026b).

b Calculations are based on a 97.5 kg carcass, 55–56% lean, and 14–18 mm of backfat thickness.

**Table 3:** Composite value realization of merchandized cuts from the loin primal (not including fixed costs).^a^^,^^b^^,^^c^

		Loin Cutting Strategy					
*Merchandized components*	Price, U.S.$/cwt	Untrimmed Loin Primal (Drop Loin)	1/4” Trimmed Loin	1/8” Trimmed Loin	Bone-in, Center-Cut, Tender-In Loin	Boneless, Center-Cut, Strap-On Loin	Boneless, Center-Cut, Strap-Off Loin
**Untrimmed loin primal (drop loin)**	85	85.00	–	–	–	–	–
**1/4” trimmed loin**	95	–	80.31	–	–	–	–
**1/8” trimmed loin**	108	–	–	87.31	–	–	–
**Bone-in, center-cut, tender-in loin**	146	–	–	–	76.08	–	–
**Boneless, center-cut, strap-on loin**	131	–	–	–	–	43.14	–
**Boneless, center-cut, strap-off loin**	160	–	–	–	–	–	46.21
**Boneless sirloin**	128	–	–	–	8.83	8.51	8.51
**Blade end**	101	–	–	–	9.84	–	–
**Tenderloin**	182	–	–	–	–	7.35	7.35
**Butt tender**	170	–	–	–	1.81	–	–
**Backribs**	264	–	–	–	–	24.10	24.10
**Riblets**	103	–	–	–	–	1.32	1.32
**72% trim**	101	–	0.44	0.44	6.28	13.06	16.53
**42% trim**	48	–	–	–	2.04	2.09	2.38
**Fat**	35	–	3.87	5.09	3.87	3.87	3.87
**Skin**	30	–	1.04	1.04	1.04	1.04	1.04
**Bone**	30	–	–	–	1.28	3.92	3.92
**Shrink**	0	–	0.00	0.00	0.00	0.00	0.00
**Total Value, U.S.%/cwt**		**85.00**	**85.67**	**93.89**	**111.07**	**108.39**	**115.23**

a Assumptions for cutting yields are derived from values reported by USDA Agricultural Marketing Service User’s Guide to USDA’s Pork Carcass Cutout (2026b).

b Calculations are based on a 97.5 kg carcass, 55–56% lean, and 14–18 mm of backfat thickness.

c Source for prices: assessed on 15 January 2026; USDA Agricultural Marketing Service Weekly National Carlot Report (2026a).

Shoulder fabrication strategies involve decisions regarding the allocation of raw material between butts and picnics, as well as their utilization in fresh, cured, or further-processed products. Processors may adjust shoulder fabrication based on relative market prices, grind demand, or export opportunities, using flexibility within this primal to balance carcass value across market conditions. Assumptions derived from the USDA for yield of merchandized cuts from the butt and picnic primals are provided in Tables 4 and 5, respectively (USDA Agricultural Marketing Service 2026b). Using single point in time data, the calculations shown in Tables 6 and 7 illustrate value captured from various butt and picnic cutting strategies, respectively. Again, these tables can be easily modified as price fluctuations occur during the year but in general, the greatest level of butt fabrication (1/4” trimmed boneless butt) has the potential to increase value proposition by approximately 6% and the greatest level of picnic fabrication (picnic meat combo, cushion out) has the potential to increase value proposition by approximately 28%.

**Table 4:** **Assumptions for cutting yields of merchandized cuts from the butt primal**.^a^^,^^b^

	Butt Cutting Strategy					
*Merchandized components*	Untrimmed Butt Primal (Drop Butt)	1/4” Trimmed Butt	1/8” Trimmed Butt	1/4” Trimmed Steak Ready Butt	1/8” Trimmed Steak Ready Butt	1/4” Trimmed Boneless Butt
**Untrimmed butt primal (drop butt)**	100.00%	–	–	–	–	–
**1/4” trimmed butt**	–	86.60%	–	–	–	–
**1/8” trimmed butt**	–	–	82.63%	–	–	–
**1/4” trimmed steak ready butt**	–	–	–	76.16%	–	–
**1/8” trimmed steak ready butt**	–	–	–	–	73.65%	–
**1/4” trimmed boneless butt**	–	–	–	–	–	78.46%
**72% trim**	–	2.31%	2.31%	12.53%	12.51%	3.69%
**42% trim**	–	–	1.24%	–	–	–
**Fat**	–	5.50%	7.96%	5.50%	7.96%	5.50%
**Skin**	–	5.09%	5.09%	5.09%	5.09%	5.09%
**Bone**	–	–	–	–	–	6.50%
**Shrink**	–	0.50%	0.77%	0.77%	0.77%	0.77%

a Assumptions for cutting yields are derived from values reported by USDA Agricultural Marketing Service User’s Guide to USDA’s Pork Carcass Cutout (2026b).

b Calculations are based on a 97.5 kg carcass, 55–56% lean, and 14–18 mm of backfat thickness.

**Table 5:** Assumptions for cutting yields of merchandized cuts from the picnic primal.^a^^,^^b^

	Picnic Cutting Strategy		
*Merchandized components*	Untrimmed Picnic Primal (Drop Picnic)	Smoker Trimmed Picnic	Picnic Meat Combo, Cushion Out
**Untrimmed picnic primal (drop picnic)**	100%	–	–
**Smoker trimmed picnic**	–	72.46%	–
**Picnic meat combo, cushion out**	–	–	49.34%
**Picnic cushion**	–	–	22.94%
**72% trim**	–	11.94%	–
**42% trim**	–	1.72%	–
**Hocks**	–	6.24%	–
**Fat**	–	3.53%	2.21%
**Skin**	–	3.66%	9.92%
**Bone**	–	–	15.15%
**Shrink**	–	0.45%	0.44%

a Assumptions for cutting yields are derived from values reported by USDA Agricultural Marketing Service User’s Guide to USDA’s Pork Carcass Cutout (2026b).

b Calculations are based on a 97.5 kg carcass, 55–56% lean, and 14–18 mm of backfat thickness.

**Table 6:** Composite value realization of merchandized cuts from the butt primal (not including fixed costs).^a^^,^^b^^,^^c^

		Butt Cutting Strategy					
Merchandized components	Price, U.S.$/cwt	Untrimmed Butt Primal (Drop Butt)	1/4” Trimmed Butt	1/8” Trimmed Butt	1/4” Trimmed Steak Ready Butt	1/8” Trimmed Steak Ready Butt	1/4” Trimmed Boneless Butt
**Untrimmed butt primal (drop butt)**	114	114.00	–	–	–	–	–
**1/4” trimmed butt**	132	–	114.31	–	–	–	–
**1/8” trimmed butt**	157	–	–	129.73	–	–	–
**1/4” trimmed steak ready butt**	149	–	–	–	113.47	–	–
**1/8” trimmed steak ready butt**	157	–	–	–	–	115.64	–
**1/4” trimmed boneless butt**	143	–	–	–	–	–	112.20
**72% trim**	101	–	2.33	2.33	12.65	12.64	3.72
**42% trim**	48	–	–	0.59	–	–	–
**Fat**	35	–	1.93	2.79	1.93	2.79	1.93
**Skin**	30	–	1.53	1.53	1.53	1.53	1.53
**Bone**	30	–	–	–	–	–	1.95
**Shrink**	0	–	–	0.00	0.00	0.00	0.00
**Total Value, U.S.%/cwt**		**114.00**	**120.10**	**136.98**	**129.58**	**132.59**	**121.32**

a Assumptions for cutting yields are derived from values reported by USDA Agricultural Marketing Service User’s Guide to USDA’s Pork Carcass Cutout (2026b).

b Calculations are based on a 97.5 kg carcass, 55–56% lean, and 14–18 mm of backfat thickness.

c Source for prices: assessed on 15 January 2026; USDA Agricultural Marketing Service Weekly National Carlot Report (2026a).

**Table 7:** Composite value realization of merchandized cuts from the picnic primal (not including fixed costs).^a^^,^^b^^,^^c^

		Picnic Cutting Strategy		
Merchandized components	Price, U.S.$/cwt	Untrimmed Picnic Primal (Drop Picnic)	Smoker Trimmed Picnic	Picnic Meat Combo, Cushion Out
**Untrimmed picnic primal (drop picnic)**	73	73.00	–	–
**Smoker trimmed picnic**	99	–	71.74	–
**Picnic meat combo, cushion out**	105	–	–	51.81
**Picnic cushion**	144	–	–	33.03
**72% trim**	101	–	12.06	–
**42% trim**	48	–	0.83	–
**Hocks**	54	–	3.37	–
**Fat**	35	–	1.24	0.77
**Skin**	30	–	1.10	2.98
**Bone**	30	–	–	4.55
**Shrink**	0	–	0.00	0.00
**Total Value, U.S.%/cwt**		**73.00**	**90.32**	**93.14**

a Assumptions for cutting yields are derived from values reported by USDA Agricultural Marketing Service User’s Guide to USDA’s Pork Carcass Cutout (2026b).

b Calculations are based on a 97.5 kg carcass, 55–56% lean, and 14–18 mm of backfat thickness.

c Source for prices: assessed on 15 January 2026; USDA Agricultural Marketing Service Weekly National Carlot Report (2026a).

Rib fabrication is often straightforward, generally consisting of trimming and improving the portion size of merchandized cuts. Belly fabrication is particularly sensitive to trim levels and thickness targets, as these directly affect bacon yield, slicing performance, and downstream processing efficiency (Person et al. 2005; Soladoye et al. 2015). Tighter specifications can improve finished product consistency and customer satisfaction but often reduce fabrication yields and increase trim volumes. Given the high economic value of ribs and bellies relative to other primals, small changes in yield or specification adherence can have outsized effects on overall carcass value. Assumptions derived from the USDA for yield of merchandized cuts from the rib and the belly primals are provided in Tables 8 and 9, respectively (USDA Agricultural Marketing Service 2026b). Using single point in time data, the calculations shown in Tables 10 and 11 illustrate value captured from various rib and belly cutting strategies, respectively. Once again, these tables can be easily modified as price fluctuations occur during the year but in general, the greatest level of rib fabrication (St. Louis spareribs) has the potential to increase value proposition by approximately 13% and the greatest level of belly fabrication (skinless belly) has the potential to increase value proposition by approximately 7%.

**Table 8:** Assumptions for cutting yields of merchandized cuts from the rib primal.^a^^,^^b^

	Rib Cutting Strategy		
*Merchandized components*	Untrimmed Rib Primal (Drop Rib)	Trimmed Sparerib	St. Louis Spareribs
**Untrimmed rib primal (drop rib)**	100%	–	–
**Trimmed spareribs**	–	95.75%	–
**St. Louis spareribs**	–	–	78.00%
**72% trim**	–	–	8.10%
**42% trim**	–	–	3.00%
**Fat**	–	3.85%	3.00%
**Bone**	–	–	7.50%
**Shrink**	–	0.40%	0.40%

a Assumptions for cutting yields are derived from values reported by USDA Agricultural Marketing Service User’s Guide to USDA’s Pork Carcass Cutout (2026b).

b Calculations are based on a 97.5 kg carcass, 55–56% lean, and 14–18 mm of backfat thickness.

**Table 9:** Assumptions for cutting yields of merchandized cuts from the belly primal.^a^^,^^b^

	Belly Cutting Strategy	
*Merchandized Components*	Natural Fall Belly (Drop Belly)	Skinless Belly
**Natural fall belly (drop belly)**	100%	–
**Skinless belly**	–	81.29%
**72% trim**	–	1.76%
**42% trim**	–	4.10%
**Fat**	–	1.59%
**Skin**	–	10.86%
**Shrink**	–	0.40%

a Assumptions for cutting yields are derived from values reported by USDA Agricultural Marketing Service User’s Guide to USDA’s Pork Carcass Cutout (2026b).

b Calculations are based on a 97.5 kg carcass, 55–56% lean, and 14–18 mm of backfat thickness.

**Table 10:** Composite value realization of merchandized cuts from the rib primal (not including fixed costs).^a^^,^^b^^,^^c^

		Rib Cutting Strategy		
Merchandized Components	Price, U.S.$/cwt	Untrimmed Rib Primal (Drop Rib)	Trimmed Sparerib	St. Louis Spareribs
**Untrimmed rib primal (drop rib)**	175	175.00	–	–
**Trimmed spareribs**	189	–	180.97	–
**St. Louis spareribs**	238	–	–	185.64
**72% trim**	101	–	–	8.18
**42% trim**	48	–	–	1.44
**Fat**	35	–	1.35	1.05
**Bone**	30	–	–	2.25
**Shrink**	0	–	0.00	0.00
**Total Value, U.S.%/cwt**		**175.00**	**182.32**	**198.56**

a Assumptions for cutting yields are derived from values reported by USDA Agricultural Marketing Service User’s Guide to USDA’s Pork Carcass Cutout (2026b).

b Calculations are based on a 97.5 kg carcass, 55–56% lean, and 14–18 mm of backfat thickness.

c Source for prices: assessed on 15 January 2026; USDA Agricultural Marketing Service Weekly National Carlot Report (2026a).

**Table 11:** Composite value realization of merchandized cuts from the belly primal (not including fixed costs).^a^^,^^b^^,^^c^

		Belly Cutting Strategy	
Merchandized components	Price, U.S.$/cwt	Natural Fall Belly (Drop Belly)	Skinless Belly
**Natural fall belly (drop belly)**	121	121.00	–
**Skinless belly**	150	–	121.94
**72% trim**	101	–	1.78
**42% trim**	48	–	1.97
**Fat**	35	–	0.56
**Skin**	30	–	3.26
**Shrink**	0	–	0.00
**Total Value, U.S.%/cwt**		**121.00**	**129.50**

a Assumptions for cutting yields are derived from values reported by USDA Agricultural Marketing Service User’s Guide to USDA’s Pork Carcass Cutout (2026b).

b Calculations are based on a 97.5 kg carcass, 55–56% lean, and 14–18 mm of backfat thickness.

c Source for prices: assessed on 15 January 2026; USDA Agricultural Marketing Service Weekly National Carlot Report (2026a).

Ham fabrication decisions are strongly influenced by end-market orientation. Bone-in hams are primarily seasonal items while boneless hams are highly coveted for value-added opportunities. Export-oriented ham programs introduce further complexity, as country-specific specifications and weight targets can alter yields and fabrication efficiency. Assumptions derived from the USDA for yield of merchandized cuts from the ham primal is provided in Table 12 (USDA Agricultural Marketing Service 2026b). Using single point in time data, the calculations shown in Table 13 illustrate value captured from various ham cutting strategies. This table can be easily modified as price fluctuations occur during the year but in general, the greatest level of ham fabrication (four-muscle ham) has the potential to increase value proposition by approximately 26%.

**Table 12:** Assumptions for cutting yields of merchandized cuts from the ham primal.^a^^,^^b^

	Ham Cutting Strategy			
*Merchandized components*	Untrimmed Ham Primal (Drop Ham)	Trimmed Selected Ham	Roll Out Ham	Four-Muscle Ham
**Untrimmed ham primal (drop ham)**	100.00%	–	–	–
**Trimmed selected ham**	–	89.70%	–	–
**Roll out ham**	–	–	53.17%	–
**Inside ham**	–	–	–	14.87%
**Outside ham**	–	–	–	16.24%
**Knuckle**	–	–	–	8.98%
**Lite butt**	–	–	–	1.67%
**Inner shank**	–	–	–	3.76%
**Outer shank**	–	–	3.55%	3.90%
**72% trim**	–	2.30%	6.96%	12.58%
**42% trim**	–	–	4.25%	6.55%
**Fat**	–	2.50%	10.20%	9.95%
**Skin**	–	5.00%	11.22%	10.97%
**Bone**	–	–	9.60%	9.60%
**Shrink**	–	0.50%	1.06%	0.95%

a Assumptions for cutting yields are derived from values reported by USDA Agricultural Marketing Service User’s Guide to USDA’s Pork Carcass Cutout (2026b).

b Calculations are based on a 97.5 kg carcass, 55–56% lean, and 14–18 mm of backfat thickness.

**Table 13:** Composite value realization of merchandized cuts from the ham primal (not including fixed costs).^a^^,^^b^^,^^c^

		Ham Cutting Strategy			
Merchandized components	Price, U.S.$/cwt	Untrimmed Ham Primal (Drop Ham)	Trimmed Selected Ham	Roll Out Ham	Four-Muscle Ham
**Untrimmed ham primal (drop ham)**	81	81.00	–	–	–
**Trimmed selected ham**	96	–	86.11	–	–
**Roll out ham**	133	–	–	70.72	–
**Inside ham**	161	–	–	–	23.94
**Outside ham**	161	–	–	–	26.14
**Knuckle**	159	–	–	–	14.28
**Lite butt**	163	–	–	–	2.72
**Inner shank**	132	–	–	–	4.96
**Outer shank**	122	–	–	4.33	4.76
**72% trim**	101	–	2.32	7.02	12.71
**42% trim**	48	–	–	2.04	3.14
**Fat**	35	–	0.88	3.57	3.48
**Skin**	30	–	1.50	3.36	3.29
**Bone**	30	–	–	2.88	2.88
**Shrink**	0	–	0.00	0.00	0.00
**Total Value, U.S.%/cwt**		**81.00**	**90.81**	**93.93**	**102.30**

a Assumptions for cutting yields are derived from values reported by USDA Agricultural Marketing Service User’s Guide to USDA’s Pork Carcass Cutout (2026b).

b Calculations are based on a 97.5 kg carcass, 55–56% lean, and 14–18 mm of backfat thickness.

c Source for prices: assessed on 15 January 2026; USDA Agricultural Marketing Service Weekly National Carlot Report (2026a).

Trim streams represent a critical, though often underappreciated, component of fabrication economics. The volume and composition of trim are directly influenced by fabrication depth and specification tightness, making trim valuation essential to accurate costing models. Lean trim supports fresh and further-processed ground products, while fat trim contributes to formulation flexibility and rendered product value. Organ meats and carcass by-products provide additional revenue streams, particularly in export-driven systems, and can significantly affect carcass valuation when effectively managed. Rendering serves as both a value recovery mechanism and a sustainability pathway, converting inedible or excess materials such as fat, skin, and bone into usable products such as tallow and protein meals (Marti et al. 2012). While rendering typically represents a lower-value outlet, its role in waste reduction and regulatory compliance makes it an integral component of comprehensive fabrication strategies.

### Trade-Offs between yield and fixed costs (ie Labor and packaging)

Deeper, more complex fabrication generally improves product specificity and market access but increases labor intensity and exposure to workforce constraints. Manual cutting remains a significant cost driver for the meat packing industry, particularly when producing portion-controlled, case-ready products. As labor costs rise or availability declines, the marginal value of additional fabrication may diminish, prompting processors to re-evaluate optimal fabrication depth.

Additionally, as fabrication depth increases, packaging requirements become more complex and influential on overall cost and product performance. Deeper fabrication often necessitates portion-sized packaging, tighter weight tolerances, and enhanced protection to preserve appearance and shelf life, particularly for case-ready products. Packaging format decisions (*e.g.*, vacuum, modified atmosphere, or overwrap) directly affect material costs, line speed, labor needs, and downstream logistics. In addition, increased use of packaging materials raises sustainability considerations and disposal costs, which may influence fabrication strategies alongside labor availability and market demands. The USDA Weekly National Carlot Report provides pricing information for different types of packaging including vacuum, paper, and combo bins (*i.e.*, high capacity bins designed to be moved by forklifts or pallet jacks) as well as price differentiations for fresh versus frozen (USDA Agricultural Marketing Service Weekly National Carlot Report, 2026a). Additionally, the User’s Guide to USDA’s Pork Carcass Cutout provides detailed pricing adjustments for merchandized products (USDA Agricultural Marketing Service 2026b).

Fixed costs in a pork packing plant include expenditures that remain relatively constant regardless of daily throughput. These costs consist of fixed overhead such as equipment investment, routine maintenance, sanitation infrastructure, utilities associated with facility operation, and depreciation of buildings and machinery. Fixed labor costs include salaried management, quality assurance, maintenance personnel, and regulatory compliance staff whose employment levels are not directly tied to production volume. Labor costs for hourly workers such as live animal handlers, slaughter floor employees, cutting floor employees, and packaging employees are generally fixed on a per carcass basis rather than a per weight basis, which will be discussed in greater detail in a subsequent section. Fixed packaging costs may include long-term contracts, packaging equipment ownership, and baseline inventory commitments that are required to maintain consistent operations, even during periods of reduced production. Additional packaging costs are likely related to depth of carcass fabrication as previously described. For the purposes of making this review comprehensive in its material, assumptions for fixed overhead (*e.g.*, equipment, maintenance, sanitation, and depreciation), fixed labor, and fixed packaging costs are presented in Table 14. The purpose of these assumptions is to provide a starting place for further calculations, and they are not meant to fit all scenarios and situations.

**Table 14:** Perceived packer opportunity of differing carcass weights when overhead and labor costs are fixed.

		Assumed Fixed Costs at Different Carcass Weights (U.S.$/kg)				
	Assumed cost	85 kg	90 kg	95 kg	100 kg	105 kg
**Fixed costs**						
** Overhead (e.g., equipment, maintenance, sanitation, depreciation, etc.), U.S.$/kg**	$14/carcass	0.1647	0.1556	0.1474	0.1400	0.1333
** Labor, U.S.$/kg**	$33/carcass	0.3882	0.3667	0.3474	0.3300	0.3143
** Packaging, U.S.$/kg**	$12/carcass	0.1412	0.1333	0.1263	0.1200	0.1143
**Total, U.S.$/kg**	**$59/carcass**	**0.6941**	**0.6556**	**0.6211**	**0.5900**	**0.5619**

Automation and mechanization increasingly influence fabrication economics by reducing labor dependency, improving consistency, and increasing throughput (Lyu et al. 2025). However, these technologies require substantial capital investment and may reduce flexibility in responding to market shifts or specification changes. As a result, the economic viability of automation depends on scale, stability of fabrication specifications, and long-term labor cost expectations. Successful fabrication strategies balance yield optimization, labor efficiency, and capital utilization to maximize overall carcass value rather than individual cut performance.

## Effect of carcass characteristics

### Carcass weight

Carcass weight is a primary determinant of fabrication efficiency, labor requirements, and product yield in commercial pork packing operations. Carcass weight directly influences line speed, ergonomic demands on labor, and the dimensional compatibility of carcasses with automated and semi-automated fabrication systems. Even so, many packing plants operate under the notion of fixed cost allocation models, treating carcasses as economically equivalent units and failing to capture the incremental labor, yield, and opportunity costs associated with weight variation. Using these fixed cost allocation models on a per carcass basis, fixed costs (*i.e.*, overhead, labor, and packaging) are reduced by meaningful magnitudes as carcasses become heavier (shown in Table 14). For example, shifting from 95 kg to 105 kg reduced fixed costs by 9.5% (U.S.$0.0592/kg). Evaluating this change for a 20,000 pig/day packing plant would equate to a perceived value capture of approximately $11,210/day (provided overhead, labor, and packaging are treated as fixed costs). The argument that overhead, labor, and packaging are not truly fixed costs should be contemplated. Heavier pigs may lead to unforeseen challenges during handling, stunning, chilling, and fabrication (Wu et al. 2017; Price et al. 2022; Zoratti et al. 2025). Furthermore, as carcass weight increases, primal and subprimal cuts generally increase in their dimensions, which can improve cutting accuracy and yield consistency but may also reduce fabrication efficiency if carcasses exceed equipment or labor design specifications.

**Figure 2:**
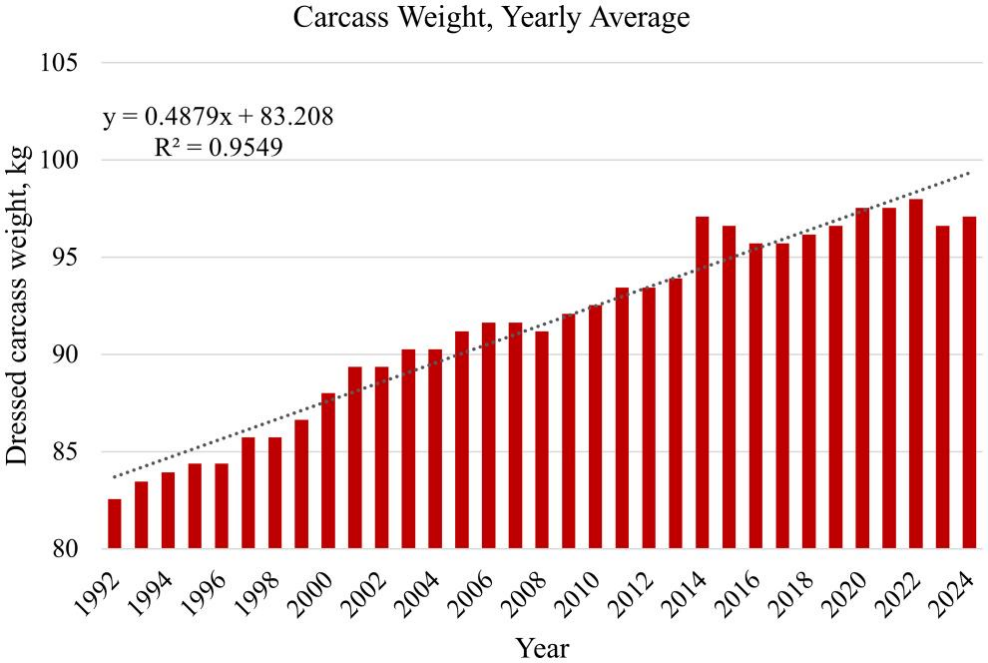
Annual carcass weights (1992–2024; source: USDA National Agricultural Statistics Service 2025).

Moderate increases in carcass weights are often associated with greater total saleable product per carcass and greater economic margins; however, excessively heavy carcasses can negatively impact labor productivity due to increased cutting resistance, handling difficulty, and worker fatigue. In addition, oversized primals may require additional trimming or rework to meet downstream product specifications, particularly in case-ready and portion-controlled programs. As a result, optimal carcass weight ranges represent a balance between maximizing yield, maintaining fabrication efficiency, and ensuring compatibility with targeted markets. An evaluation of yearly averages for carcass weight (as shown in Fig. 2) clearly illustrates that the industry has opted for moderate increases in carcass weights over the previous three decades with a linear annual increase of 0.49 kg since the year 1992.

From a market perspective, packers typically define preferred carcass weight ranges that align with customer specifications for primal dimensions, portion weights, and packaging formats. Carcasses falling outside these ranges typically experience discounted value due to reduced fabrication efficiency, lower product uniformity, or limited marketability. Consequently, carcass weight management is a critical interface between live production decisions and downstream processing economics.

### Carcass composition and yield

Beyond weight alone, carcass composition–commonly characterized by backfat thickness and muscling–plays a central role in determining fabrication yield and trim generation. Leaner carcasses with greater muscling generally produce greater proportions of saleable lean cuts, improving overall yield and value recovery (Pringle and Williams 2001). However, extremely lean carcasses (defined as carcasses with less than 12 mm of backfat thickness assessed at an optical probe grading site) may inhibit the ability to merchandize some cuts such as bone-in loins or skinless bellies, thus generating greater volumes of lean trim and potentially shifting value from whole-muscle cuts to ground or formulated products. Specifically, automated loin pullers and belly skinning equipment that are often utilized by large-volume processors have a fat thickness tolerance between 12 mm to 15 mm before lean is scored and cuts are devalued. Thus, backfat thickness influences both primal cut appearance and trimming requirements. Excessive fat deposition increases trim losses and reduces lean recovery, while insufficient fat cover can compromise fabrication. Thus, yield outcomes reflect a tradeoff between lean recovery, trim volume, and the distribution of value across product streams. These tradeoffs are particularly important in facilities that rely on trim-based products, such as sausage or further-processed items, where trim availability may offset reduced whole-muscle yields.

### Influences of carcass characteristics

**Figure 3:**
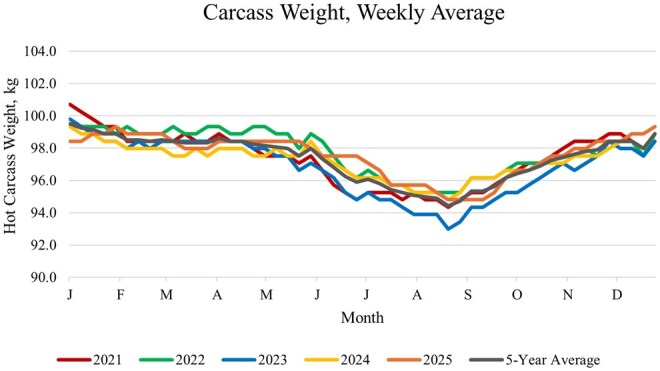
Weekly carcass weights for the most recent 5-year period (2021–2025; source: USDA agricultural marketing service estimated weekly meat production under federal inspection, 2026d).

Carcass characteristics are shaped by a complex interaction of biological and management factors, including sex, genetics, nutrition, seasonality, and other production practices. Sex differences influence growth patterns, fat deposition, and muscle distribution–with barrows, gilts, boars, and immunocastrated males exhibiting distinct differences in carcass characteristics (Boler et al. 2014; Bohrer et al. 2023a; Lei et al. 2023). Genetic selection has further amplified variation in carcass composition, as modern lines are optimized to differing levels to prioritize lean growth efficiency, muscling, and feed conversion or meat quality parameters such as color and marbling (Khanal et al. 2019; Lozada-Soto et al. 2022; Xie et al. 2023). Nutritional strategies directly affect growth rate, fat accretion, and muscle development, thereby influencing both carcass weight and composition (Lebret 2008; Yan et al. 2023). Seasonal effects, such as heat stress, may alter feed intake and growth efficiency, leading to increased variability in carcass characteristics (Renaudeau et al. 2012; Johnson and Stewart 2025). Seasonal effects are perhaps best illustrated by evaluating weekly carcass weights in the United States, which consistently decrease by 4–6 kg during the summer months of July, August and September (Fig. 3). Additional management factors, including health status, stocking density, and marketing strategy, also contribute to carcass uniformity and predictability (Arkfeld et al. 2017; Čobanović et al. 2023; Kuberka et al. 2024; Samuel et al. 2025) – all of which are critical for maintaining consistent fabrication performance in high throughput packing environments**.** 

## Influence of technological advancements

### Instrument grading

Advances in instrument grading technologies have substantially improved the ability of pork packing plants to objectively evaluate carcass composition and predict fabrication yield (López-Campos et al. 2019; Čandek-Potokar et al. 2024). Optical grading probes have been used to measure backfat thickness and muscle depth for decades (Kempster et al. 1985; Cook et al. 1989; Pomar and Marcoux 2003), and these values have been used to estimate lean composition of the carcass with varying degrees of precision (Schinckel et al. 2010; Bohrer et al. 2023b). Vision-based systems, including optical cameras, imaging technologies, and advanced in-line ultrasound technologies (such as the AutoFom III; Kolding, Denmark), are now widely available across the industry to estimate lean percentage, fat depth, and muscle dimensions at targeted grading sites or at the individual primal level (Leighton et al. 2022; Dorleku et al. 2023; Mishra and Font-i-Furnols 2024). These systems enable more accurate yield prediction compared with traditional grading techniques, providing an accurate quantitative foundation for fabrication decision-making.

Beyond yield estimation, instrument grading technologies facilitate carcass sorting into differentiated fabrication pathways. By directing carcasses toward fabrication strategies optimized for weight, dimensions, composition, or market destination, processors can improve overall value recovery and reduce inefficiencies associated with “one size fits all” fabrication models. Such sorting capabilities are increasingly important as packing plants handle wider ranges of carcass weights and compositions while serving diverse markets.

### Automation and robotics

Automation and robotics have emerged as critical tools for improving fabrication efficiency and mitigating labor constraints in modern pork packing facilities. Automated cutting and deboning systems are designed to perform repetitive, high-precision tasks with greater consistency than manual labor, particularly for standardized cuts and high-volume product streams (Delmore 2022; Kim et al. 2023; Lyu et al. 2025). When properly calibrated to recognize differences in carcass dimensions and composition, these systems can greatly enhance yield uniformity and reduce variability in trim generation (Barbar et al. 2022; Echegaray et al. 2022; Mor et al. 2022).

However, the economic justification for automation depends on a careful balance between capital investment and labor savings. High upfront costs, equipment maintenance, and integration requirements must be weighed against reductions in labor dependency, improved throughput, and enhanced worker safety (Mindlin et al. 2021; Wijaya 2025, André-Zarna et al. 2026). As labor availability becomes increasingly constrained, automation is often evaluated not solely on cost minimization, but also on risk reduction and long-term operational resilience.

### Data integration and support

The integration of real-time carcass data into fabrication operations represents a growing opportunity to optimize processing economics. Data streams from instrument grading, fabrication performance metrics, and market pricing can be combined to inform dynamic fabrication decisions at the carcass level (Echegaray et al. 2022; Hamill et al. 2024; Nayeem et al. 2025). Such approaches enable processors to align fabrication strategies with current market conditions, labor availability, and plant capacity. Emerging concepts such as digital twins and predictive modeling extend these capabilities by simulating and forecasting fabrication outcomes under alternative scenarios (Krupitzer et al. 2022; Isuru et al. 2023). Digital replicas of processing lines allow processors to evaluate how changes in carcass characteristics, line speed, or labor allocation affect yield and cost outcomes before implementation. These tools support proactive decision-making and may reduce economic risk associated with variability and market uncertainty.

## Risk, variability, and market volatility

### Price volatility of primals and subprimals

Volatility in primal and subprimal prices introduces significant uncertainty into fabrication economics. Rapid shifts in demand, and inherently value, of merchandized cuts can alter the optimal fabrication strategy, requiring packing plants to balance flexibility with operational stability. Plants with rigid fabrication systems may be unable to quickly respond to market signals, potentially forfeiting value during periods of price divergence among merchandized cuts. Flexible fabrication strategies, supported by modular processing lines and data-driven decision tools, allow packing plants to redirect product flows in response to changing market conditions. However, increased flexibility often comes at the expense of higher operational complexity, underscoring the tradeoffs between economic responsiveness and production efficiency.

### Biological variability

Biological variability in carcass weight, composition, and muscling represents a persistent source of economic risk for pork processors. A study aiming to characterize variability of pork carcass composition and primal quality reported 93.5% of the variation in carcass weight was attributed to uncontrolled biological variability while sex (barrow or gilt), seasonality, marketing group, and production focus (lean-growth or meat quality) combined to represent the other 6.5% of variation in carcass weight (Arkfeld et al. 2017). While factors such as sex (barrow or gilt) and production focus (lean-growth or meat quality) contributed to greater levels of variability for backfat thickness and iodine value (*i.e.*, fat quantity and quality), uncontrolled biological variability still accounted for 51.2% of the variation for backfat thickness and 71.9% of the variation for iodine value.

Non-uniform carcasses challenge standardized fabrication systems, increasing labor demands, trimming losses, and yield variability. Even within narrowly defined market weight ranges, heterogeneity in carcass composition can lead to meaningful differences in value recovery. Managing biological variability requires coordination across the supply chain, from genetic selection and nutritional management to marketing strategies that promote carcass uniformity. From a fabrication perspective, technologies that improve carcass characterization and sorting can partially mitigate variability-related risks, but inherent biological differences remain a fundamental constraint.

### Supply chain disruptions

Recent supply chain disruptions have highlighted the vulnerability of fabrication economics to external shocks, including labor shortages, capacity constraints, and evolving regulatory requirements (Whitehead and Kim 2022; Acosta et al. 2023). Labor availability remains a critical limiting factor for fabrication throughput, particularly in labor-intensive cutting and deboning operations. Regulatory pressures related to worker safety, line speed, and sanitation further influence operational flexibility and cost structures.In this context, fabrication strategies increasingly prioritize resilience alongside efficiency. Investments in automation, cross-trained labor, and adaptable processing systems may reduce short-term profitability but enhance long-term economic stability. As a result, risk management has become an integral component of fabrication decision-making in modern pork packing operations.

## Additional considerations

### Sustainability and efficiency considerations

Fabrication efficiency is increasingly evaluated not only in terms of economic performance but also with respect to sustainability outcomes across the pork supply chain. Several reviews have been conducted on sustainability of the pork supply chain with the production sector primarily highlighted and only surface level assessments of the packer and processor sectors (Labrecque et al. 2015; Reckmann and Krieter, 2015; Caccialanza et al. 2023). Improved fabrication efficiency can reduce losses by minimizing excessive trimming, rework, and product downgrades, thereby increasing the proportion of each carcass converted into merchandized product. Reductions in waste at the fabrication stage also decrease downstream disposal, rendering, and transportation requirements, contributing to overall resource efficiency. In the future, greater consideration for the packing and processor sector should be prioritized when evaluating sustainability of the pork supply chain.

Labor welfare and ergonomic impacts represent another critical dimension of sustainable fabrication systems. Carcass weight, line speed, and cut complexity directly influence worker fatigue, injury risk, and job satisfaction. Fabrication strategies that reduce repetitive motions, heavy lifting, or awkward postures can enhance labor welfare and sustainability while also supporting consistent productivity. As workforce availability continues to constrain processing capacity, ergonomic considerations, optimization of task design, and automation are increasingly linked to operational resilience.

Depth of fabrication also carries important environmental implications. Decisions related to cut specifications, trim management, and further processing pathways influence energy use, water consumption, and greenhouse gas emissions on a per unit of finished product basis. Fabrication systems that maximize yield efficiency and reduce reprocessing can lower the environmental footprint of pork production by improving the conversion of biological inputs into edible output. Consequently, fabrication economics and environmental performance are closely interconnected, reinforcing the need for integrated evaluation frameworks.

### Knowledge gaps and future research needs

Despite the central role of fabrication in determining pork value, publicly available data on fabrication yields, labor requirements, and cost structures remain limited. Much of the relevant information is proprietary or held by companies due to legal concerns related to Anti-Trust activity, restricting the ability of researchers to evaluate system-level tradeoffs or benchmark fabrication strategies across systems and regions. This lack of transparency constrains model development and limits the generalizability of published findings.

Future research would benefit from the development of integrated biological-economic models that link live animal characteristics with fabrication performance, labor utilization, and market outcomes. Such models could improve understanding of nonlinear relationships among carcass weight, composition, and processing efficiency, while also capturing interactions with price volatility and capacity constraints. Advances in data collection and analytics offer opportunities to move beyond static assumptions toward dynamic, decision-support tools.

Opportunities for collaboration between industry partners are essential to address these gaps. Partnerships that facilitate controlled data sharing, pilot-scale experimentation, and validation of modeling approaches can accelerate progress while respecting commercial sensitivities. By aligning research objectives with practical industry challenges, collaborative efforts can generate actionable insights that enhance both scientific understanding and the economic and environmental sustainability of pork fabrication systems.

## Conclusions

Pork carcass fabrication represents a critical nexus where biological variation, market signals, and processing constraints converge to determine realized carcass value. As outlined in this review, fabrication decisions, such as depth of fabrication, cut specifications, and allocation of primals, subprimals, trim, and by-products, play a central role in translating carcass characteristics into economic outcomes for packers, processors, and integrated production systems. Within an industry characterized by narrow margins and high throughput, small changes in yield, labor efficiency, or specification adherence can result in disproportionately large impacts on profitability.

The economic value of pork carcasses is not inherent but is actively shaped through fabrication strategies that respond to regional market preferences, pricing systems, and end-user demands. Differences in cutting specifications across global markets, coupled with volatility in primal and subprimal prices, underscore the importance of flexibility and market awareness in fabrication planning. At the same time, carcass weight, composition, and uniformity strongly influence fabrication efficiency, labor requirements, and trim generation, reinforcing the need for alignment between production decisions and downstream processing capabilities.

Technological advancements, such as instrument grading, automation, and data integration, are increasingly re-defining how fabrication decisions are made and executed. Objective carcass assessment and real-time sorting enable more targeted fabrication strategies, while automation offers pathways to mitigate labor constraints and improve consistency. Emerging tools such as predictive modeling and digital twins further extend these capabilities by allowing processors to evaluate alternative scenarios and manage risk in an environment marked by biological variability and market uncertainty. However, the economic viability of these technologies depends on scale, capital availability, and the stability of fabrication specifications, highlighting the need for balanced and defined investment strategies.

Importantly, fabrication economics extend beyond yield maximization to encompass labor welfare, sustainability, and operational resilience. Decisions related to carcass weight targets, fabrication depth, and packaging formats have implications for worker ergonomics, environmental footprint, and long-term system efficiency. As supply chain disruptions and workforce challenges persist, fabrication strategies increasingly prioritize long-term robustness and adaptability alongside short-term value capture.

Collectively, the evidence reviewed here emphasizes that optimal pork carcass fabrication is not defined by a single set of specifications or practices, but rather by the strategic integration of biological inputs, economic signals, and operational constraints. Improved coordination across the supply chain, coupled with greater transparency and data-driven decision-making, offers substantial opportunities to enhance value realization. Continued collaboration between industry partners will be essential to develop integrated biological-economic frameworks that support more efficient, resilient, and sustainable pork fabrication systems in the future.

## Abbreviations:

IMPS: Institutional Meat Purchase Specifications
UNECE: United Nations Economic Commissions for Europe
USDA: United States Department of Agriculture

## References

[txag024-B1] Acosta A. , Lloyd T., McCorriston S., Lan H. 2023. The ripple effect of animal disease outbreaks on food systems: the case of African Swine Fever on the Chinese pork market. Prev. Vet. Med. 215:105912. 10.1016/j.prevetmed.2023.105912 37119649

[txag024-B2] André-Zarna C. , Simmen K., Albretsen K. H., Berg P., Njaastad E. B. 2026. Meat industry 5.0–a review of technological approaches and robotic systems in the meat processing industry. Trends Food Sci. Technol. 167:105439. 10.1016/j.tifs.2025.105439

[txag024-B3] Arkfeld E. K. et al 2017. Characterization of variability in pork carcass composition and primal quality. J. Anim. Sci. 95:697–708. 10.2527/jas.2016.1097 29432540

[txag024-B4] Australian Pork. 2023. Pork training manual. https://australianpork.com.au/sites/default/files/2023-01/Pork%20training%20manual%20FINAL.pdf

[txag024-B5] Barbar C. , Bass P. D., Barbar R., Bader J., Wondercheck B. 2022. Artificial intelligence-driven automation is how we achieve the next level of efficiency in meat processing. Anim. Front. 12:56–63. 10.1093/af/vfac017 PMC9056041 35505849

[txag024-B6] Bohrer B. M. , Dorleku J. B., Campbell C. P., Duarte M. S., Mandell I. B. 2023a. A comparison of carcass characteristics, carcass cutting yields, and meat quality of barrows and gilts. Transl. Anim. Sci. 7:txad079. 10.1093/tas/txad079 37649648 PMC10464715

[txag024-B7] Bohrer B. M. , Wang Y., Dorleku J. B., Campbell C. P., Mandell I. B. 2023b. An update of the predicted lean yield equation for the Destron PG-100 optical grading probe. J. Anim. Sci. 101:skad199. 10.1093/jas/skad199 37317891 PMC10313092

[txag024-B8] Boland M. A. , Foster K. A., Akridge J. T. 1995. Packer sorting strategies for fresh pork. Agribusiness. 11:423–430. 10.1002/1520-6297(199509/10)11:5%3C423::AID-AGR2720110505%3E3.0.CO; 2-X

[txag024-B9] Boler D. D. et al 2014. Effects of immunological castration (Improvest) on changes in dressing percentage and carcass characteristics of finishing pigs. J. Anim. Sci. 92:359–368. 10.2527/jas.2013-6863 24243892

[txag024-B10] Bryan E. E. , Overholt M. F., Kim G. D., Dilger A. C., Boler D. D. 2018. Evaluation of alternative fabrication specifications to increase gross value of pork carcasses. Transl. Anim. Sci. 2:19–25. 10.1093/tas/txy003 32704686 PMC7200862

[txag024-B11] Caccialanza A. , Cerrato D., Galli D. 2023. Sustainability practices and challenges in the meat supply chain: a systematic literature review. Br. Food J. 125:4470–4497. 10.1108/BFJ-10-2022-0866

[txag024-B12] Čandek-Potokar M. , Lebret B., Gispert M., Font-I-Furnols M. 2024. Challenges and future perspectives for the european grading of pig carcasses–a quality view. Meat Sci. 208:109390. 10.1016/j.meatsci.2023.109390 37977057

[txag024-B13] Cannon J. E. et al 1995. Pork quality audit: a review of the factors influencing pork quality. J. Muscle. Food. 6:369–402. 10.1111/j.1745-4573.1995.tb00581.x

[txag024-B14] Čobanović N. , Suvajdžić B., Vićić I., Vasilev D., Karabasil N. 2023. Prevalence of carcass lesions and their effects on welfare, carcass composition and meat quality in slaughtered pigs. Annals Anim. Sci. 23:597–609. 10.2478/aoas-2022-0093

[txag024-B15] Cook G. L. , Chadwick J. P., Kempster A. J. 1989. An assessment of carcass probes for use in Great Britain for the EC pig carcass grading scheme. Anim. Sci. 48:427–434. 10.1017/S0003356100040423

[txag024-B16] Delmore R. J. 2022. Automation in the global meat industry. Anim. Front. 12:3–4. 10.1093/af/vfac021

[txag024-B17] Dorleku J. B. et al 2023. Comparison of an advanced automated ultrasonic scanner (AutoFom III) and a handheld optical probe (Destron PG-100) to determine lean yield in pork carcasses. J. Anim. Sci. 101:skad058. 101093/jas/skad058 36807699 10.1093/jas/skad058 PMC10032186

[txag024-B18] Echegaray N et al 2022. Meat 4.0: principles and applications of industry 4.0 technologies in the meat industry. Appl. Sci. 12:6986. 10.3390/app12146986

[txag024-B19] Hamill, R. M. et al. 2024. Toward meat industry 4.0: Opportunities and challenges for digitalized red meat processing. In A. Hassoun, editor, Food Industry 4.0. Academic Press, Cambridge, Massachusetts. p. 259–281.

[txag024-B20] Hayenga M. L. , Grisdale B. S., Kauffman R. G., Cross H. R., Christian L. L. 1985. A carcass merit pricing system for the pork industry. Am. J Agric. Econ. 67:315–319. 10.2307/1240684

[txag024-B21] Interporc Spain. 2020. The guide to meats in Spain – Pork. https://www.interporcspain.org/uploads/1/2/0/5/120592379/guide_to_meats_in_spain_eng_.pdf

[txag024-B22] Isuru A. , Kelton W., Bayer C. 2023. Digital twins in food processing: a conceptual approach to developing multi-layer digital models. Digit. Chem. Eng. 7:100087. 10.1016/j.dche.2023.100087

[txag024-B23] Johnson J. S. , Stewart K. R. 2025. Heat stress matters: insights from United States swine producers. Transl. Anim. Sci. 9:txaf001. 10.1093/tas/txaf001 39906722 PMC11792652

[txag024-B24] Keeler G. L. , Tokach M. D., Goodband R. D., Nelssen J. L. 1994. Assisting swine producers to maximize marketing returns. J. Ext. 32:32. https://commons.joe.org/joe/vol32/iss1/32/

[txag024-B25] Kempster A. J. , Chadwick J. P., Jones D. W. 1985. An evaluation of the hennessy grading probe and the SFK Fat-O-Meater for use in pig carcass classification and grading. Anim. Sci. 40:323–329. 10.1017/S0003356100025447

[txag024-B26] Khanal P. , Maltecca C., Schwab C., Gray K., Tiezzi F. 2019. Genetic parameters of meat quality, carcass composition, and growth traits in commercial swine. J. Anim. Sci. 97:3669–3683. 10.1093/jas/skz247 31350997 PMC6735811

[txag024-B27] Kim J. , Kwon Y.-K., Kim H. W., Seol K. H., Cho K. 2023. Robot technology for pork and beef meat slaughtering process: a review. Animals. 13:651. 10.3390/ani13040651 36830438 PMC9951719

[txag024-B28] Krupitzer C. , Noack T., Borsum C. 2022. Digital food twins combining data science and food science: system model, applications, and challenges. Processes. 10:1781. 10.3390/pr10091781

[txag024-B29] Kuberka Z et al 2024. Relationships between pig farm management and facilities and lung lesions’ scores and between lung lesions scores and carcass characteristics. BMC Vet. Res. 20:124. 10.1186/s12917-024-03968-2 38539145 PMC10976837

[txag024-B30] Labrecque J. , Dulude B., Charlebois S. 2015. Sustainability and strategic advantages using supply chain-based determinants in pork production. Br. Food J. 117:2630–2648. 10.1108/BFJ-02-2015-0068

[txag024-B31] Lebret B. 2008. Effects of feeding and rearing systems on growth, carcass composition and meat quality in pigs. Animal. 2:1548–1558. 10.1017/S1751731108002796 22443914

[txag024-B32] Lei X. I. E. , Lin R. A. O., Deng-Shuai C. U. I., Xi T. A. N. G., Shi-Jun X. I. A. O. 2023. Effects of carcass weight, sex and breed composition on meat cuts and carcass trait in finishing pigs. J. Integr. Agric. 22:1489–1501. 10.1016/j.jia.2022.08.122

[txag024-B33] Leighton P. L. A. et al 2022. Prediction of carcass composition and meat and fat quality using sensing technologies: a review. Meat Muscl. Bio. 5:1–21. 10.22175/mmb.12951

[txag024-B34] López-Campos Ó. , Prieto N., Juárez M., Aalhus J. L. 2019. New technologies available for livestock carcass classification and grading. CABI Rev. 2019:1–10. 10.1079/PAVSNNR201914018

[txag024-B35] Lorenzen C. L. et al 1996a. Subprimal purchasing and merchandising decisions for pork: Relationship to retail yield and fabrication time. J. Anim. Sci. 74:5–12. 10.2527/1996.7415 8778112

[txag024-B36] Lorenzen C. L. et al 1996b. Subprimal purchasing and merchandising decisions for pork: Relationship to retail value. J. Anim. Sci. 74:13–17. 10.2527/1996.74113x 8778091

[txag024-B37] Lozada-Soto E. A. et al 2022. Genotyping and phenotyping strategies for genetic improvement of meat quality and carcass composition in swine. Genet. Sel. Evol. 54:42. 10.1186/s12711-022-00736-4 35672700 PMC9171933

[txag024-B38] Lyu Y et al 2025. A review of robotic and automated systems in meat processing. Front. Robot. AI. 12:1578318. 10.3389/frobt.2025.1578318 40485769 PMC12141337

[txag024-B39] Marti D. L. , Johnson R. J., Mathews K. H. Jr. 2012. Where’s the (not) meat? Byproducts from beef and pork production. J. Curr. Issues Glob. 5:397–423.

[txag024-B40] Metz J. L. et al 2024. Influence of increasing carcass weights on pork carcass characteristics and traditional and alternative fabrication yields. Meat Muscl. Biol. 8:16304. 10.22175/mmb.16304

[txag024-B41] Mindlin Y. B. , Gorbunova A. O., Tarasova T. M., Korobeynikova E. V., Filimoshina O. V. 2021. Economic efficiency of introduction of additive technologies in the meat processing industry. In IOP Conf. Ser. Earth Environ. Sci. 839:022062. 10.1088/1755-1315/839/2/022062

[txag024-B42] Mishra P , Font‐i‐Furnols M. 2024. X‐ray computed tomography meets robust chemometric latent space modeling for lean meat percentage prediction in pig carcasses. J. Chemom. 38:e3591. 10.1002/cem.3591

[txag024-B43] Mor , Kumar R. S. D., Singh A., Neethu K. 2022. Robotics and automation for agri-food 4.0: Innovation and challenges. In: Agri-food 4.0: Innovations, challenges and strategies. Leeds, England: Emerald Publishing Limited. p. 189–199. 10.1108/S1877-636120220000027013

[txag024-B44] Nayeem M. , Rahman M. H., Rahman M. A., Haque M. N. H., Hashem M. A. 2025. Advancing the meat industry with machine learning: a study of progress, challenges, and potential. Meat Res. 5:113. 10.55002/mr.5.2.113

[txag024-B45] Person R. C. et al 2005. Benchmarking value in the pork supply chain: processing characteristics and consumer evaluations of pork bellies of different thicknesses when manufactured into bacon. Meat Sci. 70:121–131. 10.1016/j.meatsci.2004.12.012 22063288

[txag024-B46] Pomar C , Marcoux M. 2003. Comparing the Canadian pork lean yields and grading indexes predicted from grading methods based on destron and hennessy probe measurements. Can. J. Anim. Sci. 83:451–458. 10.4141/A02-107

[txag024-B47] Price H. E. et al 2022. Differences in carcass chilling rate underlie differences in sensory traits of pork chops from pigs with heavier carcass weights. J. Anim. Sci. 100:skac206. 10.1093/jas/skac206 35727741 PMC9412177

[txag024-B48] Pringle T. D. , Williams S. E. 2001. Carcass traits, cut yields, and compositional end points in high-lean-yielding pork carcasses: effects of 10th rib backfat and loin eye area. J Anim Sci. 79:115–121. 10.2527/2001.791115x 11204691

[txag024-B49] Ray F. K , Cravens J. W. 2002. International pork specifications. Proceedings of the 2002 Reciprocal Meats Conference; American Meat Science Association, Kearney, Missouri. https://meatscience.org/docs/default-source/publications-resources/rmc/2002/international-pork-specifications(2).pdf? sfvrsn=2

[txag024-B50] Reckmann K , Krieter J. 2015. Environmental impacts of the pork supply chain with regard to farm performance. J. Agric. Sci. 153:411–421. 10.1017/S0021859614000501

[txag024-B51] Renaudeau , Gilbert D. H., Noblet J. 2012. Effect of climatic environment on feed efficiency in swine. In: Feed efficiency in swine. Wageningen Academic, Wageningen, Netherlands. p. 183–210. 10.3920/9789086867561_011

[txag024-B52] Samuel R. S. , Darrington J. E., St-Pierre B., Levesque C. L., Thaler R. C. 2025. Effect of pen space allowances on growth performance of finishing pigs. Animals. 15:1451. 10.3390/ani15101451 40427328 PMC12108397

[txag024-B53] Schinckel A. P. , Wagner J. R., Forrest J. C., Einstein M. E. 2010. Evaluation of the prediction of alternative measures of pork carcass composition by three optical probes. J. Anim. Sci. 88:767–794. 10.2527/jas.2009-2286 19820040

[txag024-B54] Seong P. N. et al 2014. Characterization of edible pork by-products by means of yield and nutritional composition. Korean J. Food Sci. Anim. Resour. 34:297–306. 10.5851/kosfa.2014.34.3.297 26761170 PMC4597865

[txag024-B55] Soladoye P. O. , Shand P. J., Aalhus J. L., Gariépy C., Juárez M. 2015. Pork belly quality, bacon properties and recent consumer trends. Can. J. Anim. Sci. 95:325–340. 10.4141/cjas-2014-121

[txag024-B56] Standardization Administration of the People’s Republic of China. 2019. GB/T 9959.3-2019: Fresh and frozen pork and pig by-products–Part 3: Pork cuts. https://www.chinesestandard.net/PDF/English.aspx/GBT9959.3-2019

[txag024-B57] The Meat Institute. 2025. The meat buyer’s guide (9th ed.). The Meat Institute and American Meat Science Association, Arlington, Virginia.

[txag024-B58] Tonsor G , Parcell J. 2025. A review of the pork cutout estimation and attributes of LMR wholesale pork used in the cutout estimation. https://www.ams.usda.gov/sites/default/files/media/AMSPorkCutoutFinalReport.pdf

[txag024-B59] UNECE. 2008. United Nations Economic Commission of Europe Standard: porcine meat – carcases and cuts (Symbol ECE/TRADE/369/Rev.3). https://unece.org/DAM/trade/agr/standard/standard/meat/e/Porcine_Rev3_2018E.pdf

[txag024-B60] USDA Agricultural Marketing Service. 2026a. Weekly National Carlot Meat Report. National weekly pork report–FOB plant, negotiated sales; Des Moines, IA: USDA‑AMS Livestock, Poultry & Grain Market News. https://mymarketnews.ams.usda.gov/filerepo/reports? field_slug_id_value=&name=&field_slug_title_value=lm_pk610&field_published_date_value=&field_report_date_end_value=&field_api_market_types_target_id=All

[txag024-B61] USDA Agricultural Marketing Service. 2026b. User’s guide to USDA’s pork carcass cutout; Des Moines, IA: USDA‑AMS Livestock, Poultry & Grain Market News. https://www.ams.usda.gov/sites/default/files/media/LMRPorkCutoutHandout.pdf

[txag024-B62] USDA Agricultural Marketing Service. 2026c. Weekly by-product drop value report. National; Des Moines, IA: USDA‑AMS Livestock, Poultry & Grain Market News. https://www.ams.usda.gov/mnreports/ams_2835.pdf

[txag024-B63] USDA Agricultural Marketing Service. 2026d. Estimated weekly meat production under federal inspection; Saint Joseph, MO: USDA‑AMS Livestock, Poultry & Grain Market News. https://mymarketnews.ams.usda.gov/viewReport/3209

[txag024-B64] USDA National Agricultural Statistics Service. 2025. Quick stats: hogs, slaughter, commercial, fi–slaughtered, measured in lb/ head, dressed basis. https://quickstats.nass.usda.gov/

[txag024-B65] Whitehead D , Kim Y. H. B. 2022. The impact of COVID 19 on the meat supply chain in the USA: a review. Food Sci. Anim. Resour. 42:762–774. 10.5851/kosfa.2022.e39 36133635 PMC9478983

[txag024-B66] Wijaya A. 2025. Analyzing return on investment models and long-term profitability of robotic arm deployments in high-volume food manufacturing operations. J. Robot. Proc. Auto., AI Integr. Work. Opti. 10:1–9.

[txag024-B67] Wu F et al 2017. A review of heavy weight market pigs: status of knowledge and future needs assessment. Transl. Anim. Sci. 1:1–15. 10.2527/tas2016.0004 PMC7235466 32704624

[txag024-B68] Xie L et al 2023. Genetic dissection of 26 meat cut, meat quality and carcass traits in four pig populations. Genet. Sel. Evol. 55:43. 10.1186/s12711-023-00817-y 37386365 PMC10311868

[txag024-B69] Yan E. , Guo J., Yin J. 2023. Nutritional regulation of skeletal muscle energy metabolism, lipid accumulation and meat quality in pigs. Anim. Nutr. 14:185–192. 10.1016/j.aninu.2023.04.009 37808951 PMC10556049

[txag024-B70] Zhou Z. Y. , Bohrer B. M. 2019. Defining pig sort loss with a simulation of various marketing options of pigs with the assumption that marketing cuts improve variation in carcass weight and leanness. Can. J. Anim. Sci. 99:542–552. 10.1139/cjas-2018-0195

[txag024-B71] Zoratti A et al 2025. Simulation of loading and unloading through ramps of different configuration: effects on the ease of handling and physiological response of pigs of two slaughter weights. Can. J. Anim. Sci. 105:1–9. 10.3390/ani13172767

